# Distinctive gut microbiomes of ankylosing spondylitis and inflammatory bowel disease patients suggest differing roles in pathogenesis and correlate with disease activity

**DOI:** 10.1186/s13075-022-02853-3

**Published:** 2022-07-07

**Authors:** P. R. Sternes, L. Brett, J. Phipps, F. Ciccia, T. Kenna, E. de Guzman, K. Zimmermann, M. Morrison, G. Holtmann, E. Klingberg, D. Mauro, C. McIvor, H. Forsblad-d’Elia, M. A. Brown

**Affiliations:** 1 Centre for Microbiome Research, Queensland University of Technology, Brisbane, Australia; 2 Department of Gastroenterology, Logan Hospital, Logan, Australia; 3 School of Biomedical Sciences, Queensland University of Technology, Brisbane, Australia; 4 Department of Precision Medicine, Università della Campania L. Vanvitelli, Naples, Italy; 5 Centre for Immunology and Infection Control, Queensland University of Technology, Brisbane, Australia; 6 School of Chemistry and Molecular Biosciences, University of Queensland, Brisbane, Australia; 7 Faculty of Health and Behavioural Sciences, University of Queensland, Brisbane, Australia; 8 Department of Rheumatology and Inflammation Research, Sahlgrenska Academy at University of Gothenburg, Gothenburg, Sweden; 9 Genomics England, London, UK; 10 Faculty of Life Sciences and Medicine, King’s College London, London, UK

**Keywords:** Ankylosing spondylitis, Inflammatory Bowel disease, Dysbiosis, Gut

## Abstract

**Background:**

Multiple studies have confirmed dysbiosis in ankylosing spondylitis (AS) and inflammatory bowel disease (IBD); however, due to methodological differences across studies, it has not been possible to determine if these diseases have similar or different gut microbiomes.

**Results:**

In this study, faecal and intestinal biopsies were obtained from 33 Australian AS patients (including 5 with concomitant IBD, ‘AS-IBD’), 59 IBD patients and 105 healthy controls. Stool samples were also obtained from 16 Italian AS patients and 136 Swedish AS patients. Focusing on the Australian cohort, AS, AS-IBD and IBD patients differed from one another and from healthy controls in both alpha and beta diversity. AS patients with and without clinical IBD could be distinguished from one another with moderate accuracy using stool microbiome (AUC=0.754). Stool microbiome also accurately distinguished IBD patients from healthy controls (AUC=0.757). Microbiome composition was correlated with disease activity measured by BASDAI and faecal calprotectin (FCP) levels. Enrichment of potentially pathogenic *Streptococcus* was noted in AS, AS-IBD and IBD patients. Furthermore, enrichment of another potentially pathogenic genus, *Haemophilus*, was observed in AS, AS-IBD, IBD, AS patients with increased BASDAI, and IBD patients with faecal calprotectin >100 μg/mg. Apart from these genera, no other taxa were shared between AS and IBD patients.

**Conclusions:**

In conclusion, the distinct gut microbiome of AS and AS-IBD patients compared to IBD patients and healthy controls is consistent with immunological and genetic evidence suggesting that the gut plays a different role in driving AS compared with IBD. However, enrichment of two potentially pathogenic genera in both diseases suggests that the presence of a shared/common microbial trigger of disease cannot be discounted.

**Supplementary Information:**

The online version contains supplementary material available at 10.1186/s13075-022-02853-3.

## Introduction

Genetics research has provided important information as to the cause of the development of both ankylosing spondylitis (AS) and inflammatory bowel disease (IBD). There are strong genetic components in the risks of developing both conditions, with high heritability of AS [1, 2] and IBD [3] demonstrated in twin studies. The high disease heritability indicates that the environmental factors involved in the disease are likely to be ubiquitous. AS is strongly associated with *HLA-B27*, which contributes roughly 20% of the disease’s heritability. The carriage of HLA alleles (including *HLA-B27*) has been correlated with microbial dysbiosis even in healthy subjects, indicating that the gut microbiome may be a driver of disease, as opposed to being secondary to it [4, 5]. There are at least 163 loci reported so far to be associated with IBD, with a strong overlap between the genes associated with AS and IBD [6].

Up to 70% of AS patients have evidence of terminal ileitis resembling IBD, suggesting gut inflammation is important in disease pathogenesis [7, 8]. Increased gut permeability has been demonstrated in AS patients consistent with the hypothesis that ‘leakiness’ to gut microbes may drive inflammation in the disease [9–12]. However, unlike ileal IBD, AS-associated terminal ileitis is very rarely trans-mural and does not cause strictures or perforation.

There is strong evidence to suggest that the gut microbiome or gut pathogens trigger the development of IBD [13]. Diversion of the faecal stream dramatically reduces the risk of disease recurrence in the neo-terminal ileum of IBD patients who have undergone ileal resection for severe disease, and antibiotics can similarly reduce the risk of post-operative recurrence of ileal disease [14, 15]. Genetic studies in IBD show a marked over-representation of genes which are also associated with the risk of developing mycobacterial diseases, including leprosy and tuberculosis [16]. Faecal microbiome transplants, correcting IBD-associated dysbiosis, are therapeutically effective in IBD [17].

To date, numerous studies have confirmed the differing microbial profiles of cases versus controls for both AS and IBD [13, 18–25]. Studies have also shown a relationship between disease activity and microbial profile [22]. Whilst mounting evidence indicates a causal role for gut microbiome in driving AS and IBD, immunological and genetic evidence implies differing modes of pathogenicity. The recent demonstration that treatment with vedolizumab, which is effective for IBD through its effect on reducing lymphocyte migration in the gut mucosa, can induce de novo axial spondyloarthritis, indicates that the mechanisms by which the gut is involved in driving IBD and AS have significant differences [26]. However, direct comparison of the AS and IBD microbiomes has not been possible due to divergence of metagenomic approaches and differences in population demographics. In this study, we collected stool samples, terminal ileal biopsies, colonic biopsies and rectal biopsies from Australian AS patients, IBD patients and healthy controls, as well as stool samples from Italian and Swedish AS patients to (1) test the hypothesis that the gut microbiome in AS and IBD is different, (2) compare the AS microbiome across different populations, and (3) investigate the relationship between AS and IBD disease activity with microbiome composition.

## Methods

### Human subjects

Ethical approval for this study was obtained from the Princess Alexandra Hospital and the Queensland University of Technology (QUT) human research ethics committees (Metro South approval no. HREC/15/QPAH/309 and QUT approval no. 1600000188), and the research ethics committees of the University of Palermo (CE number 5/2014 16042014) and Sahlgrenska University Hospital in Gothenburg (CE numbers 597-08 and 690 13). For the Australian and Italian cohorts, patients were recruited with written informed consent from rheumatology and gastroenterology endoscopy outpatient departments (Princess Alexandra Hospital (Australia), Logan Hospital (Australia), University of Palermo (Italy)), and Sahlgrenska University Hospital, Borås Hospital and Alingsås Hospital, Sweden. An overview of the sampled cohorts is available in Table 1. AS was in each case defined according to the modified New York AS criteria [27].

**Table 1:** Demographics of the sampled cohorts

	Australia				Italy	Sweden
	AS	AS-IBD	IBD	HC	AS	AS
**Age (mean ± SD)**	48.5 ± 15.3	60 ± 9.8	42.7 ± 11.6	62.2 ± 10.3	39.6 ± 10.1	55.8 ± 11.8
**BMI (mean ± SD)**	26.9 ± 5.5	26.2 ± 3.3	27.7 ± 7.0	27.3 ± 6.1	27.6 ± 4.8	27.72 ± 5.7
**Gender (% male)**	86	60	42	54	69	70
**Ethnicity (% white)**	100	100	94	77	100	100
**No. individuals**	28	5	59	105	16	136
**No. samples**	52	15	199	311	16	211
**% NSAID**	50	50	5	17	31	57
**% DMARD**	18	17	72	0	0	76
**% TNF**	84	83	45	0	0	100
**% steroid**	14	33	13	3	n/a	4

For all Australian patients (AS, AS-IBD, IBD and HC), biopsies from the terminal ileum, rectum and right colon were acquired from patients undergoing routine colonoscopy, with samples gathered from neighbouring inflamed and non-inflamed tissue collected where possible. Stool samples were collected from the Australian and Italian cohorts. All samples were snap-frozen and stored at −80°C (mucosal biopsies) or −30°C (stool samples) prior to processing.

Overall, the Australian cohort consisted of 28 AS patients, 5 AS patients with concomitant IBD, 59 IBD patients and 105 healthy controls. The Italian cohort consisted of 16 AS patients. Stool samples from 136 AS patients, some sampled twice at a five-year interval, as part of the Swedish cohort were collected as previously described [25] and shipped frozen to Australia for DNA extraction and processing.

Across all cohorts, the participants were predominantly of Caucasian descent and followed an omnivorous diet. Patients with recent (6 months) antibiotic or probiotic usage were excluded from further analysis. Samples across all three cohorts were collected and processed at the same time using standardised techniques, as described below.

For all AS patients, disease activity was fully assessed by blood tests (full blood count, CRP, and ESR), and patient-reported disease activity (BASDAI). Bowel symptoms were evaluated quantitatively in all patients with AS using the spondyloarthritis modification of the Dudley Inflammatory Bowel Symptom Questionnaire (DISQ), a validated outcome measure described previously [28]. Faecal calprotectin was used as an objective marker of bowel inflammation as was measured using ELISA as described previously [28].

### DNA extraction

Samples were thawed to room temperature at time of DNA extraction. DNA was extracted from human stool samples using the Promega Maxwell® 16 LEV Blood DNA Kit (#AS1290) according to the manufacturer’s instructions. At the completion of DNA extraction RNase was added and incubated at 37 °C for 15 min, before RNA concentration was checked by Nanodrop.

### 16S sequencing prep

Samples were quantified using Agilent 4200 TapeStation and normalised to 50 ng/μL for a total of 200ng per sample DNA (4 μL per sample). First stage PCR was performed using 517F and 803R regions of the 16S rRNA gene at a 25 μL half-reaction volume for 25 PCR cycles [19]. A QC step of Agilent 4200 TapeStation analysis to confirm amplification size was performed before Beckman AMPure XP magnetic bead clean-up protocol was completed. Dual Indexing PCR was performed using the Nextera XT indexing kits and the manufacturer’s protocol. Samples were then pooled using the Perkin Elmer JANUS Liquid Handler running the “normalisation” protocol which automatically randomised samples to eliminate batch effect. Agilent 4200 TapeStation QC was performed to confirm a concentration of ~2 nM before the library was denatured with 10% PhiX.

### Data processing of 16S gene amplicons

FASTQ files were interrogated using FastQC v0.11.5 [29] and samples with low per base sequencing qualities were removed. FASTQ files were trimmed and the depth was assessed using QIIME2 v2020.2 [30]. Samples with low amplicon abundance (<4485 amplicons) were excluded from further analysis. Subsequently, reads were joined using PEAR v0.9.10 [31] and imported, quality filtered, denoised, classified (Greengenes; gg_13_8, 99% similarity), and exported at the genus level using QIIME2.

### Statistical analysis

Multidimensional data visualisation of beta diversity was conducted using a sparse partial least squares discriminant analysis (sPLSDA) on arcsine squared root transformed data, as implemented in R v3.5.2 [32] as part of the MixOmics package v6.3.1 [33]. Association of the microbial composition (beta diversity) with metadata of interest was conducted using a PERMANOVA test as part of vegan v2.4-5 [34] on arcsine square-root transformed data at the genus level, taking into account individual identity where multiple sites per individual were co-analysed, as well as the sources of covariation such as BMI, ethnicity, age, gender, smoking status and TNFi usage. Alpha diversity was calculated at the species level using the rarefy function as implemented in vegan v2.4-5 and differences were evaluated using a Wilcoxon rank-sum test. Differential abundances of bacterial taxa were tested for significance using MaAsLin2 v0.2.3 [20]. Graphs and figures were generated using ggplot2 v3.3.2 [35].

## Results

**Fig. 1:**
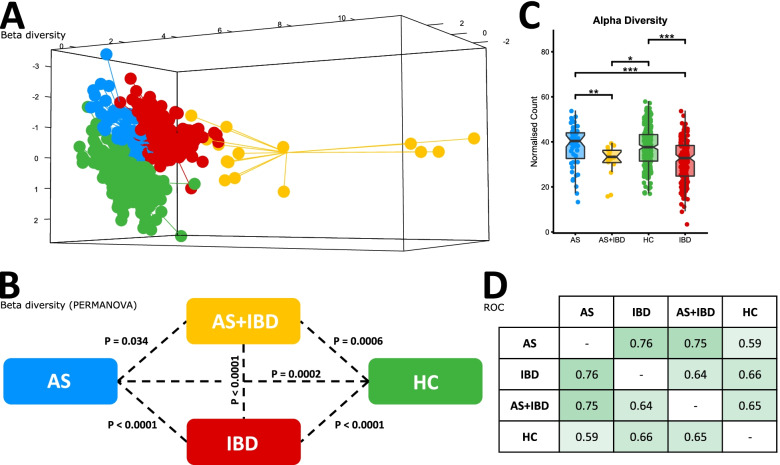
Comparison of AS patients, IBD patients, AS patients with concomitant IBD, and healthy controls in the Australian cohort, sampled from the terminal ileum, rectum, right colon and stool, whilst accounting for repeated sampling. **A** sPLSDA visualisation of overall microbiome composition (beta diversity). **B** PERMANOVA significance testing of beta diversity. **C** Comparison of species richness (alpha diversity). **D** ROC analysis for the detection of IBD in the stools of AS patients

It has been well established that the gut microbiome of AS and IBD patients are significantly different to healthy controls. However, it is less clear whether the AS and IBD microbiomes differ from each other. Utilising a combination of stool samples and biopsies of the terminal ileal, colonic and rectal mucosae collected from 33 AS patients (including 5 with concomitant IBD, ‘AS-IBD’), 59 IBD patients, and 105 controls, significant differences in beta diversity between AS patients and healthy controls, as well as IBD patients and healthy controls were reconfirmed (Fig. 1A, B). Also consistent with previous results [5, 24] the alpha diversity of the microbiome of AS patients was the same as healthy controls, whereas IBD patients exhibited a significant depletion of bacterial diversity (Fig. 1C). AS patients with concomitant IBD (‘AS-IBD’) also exhibited a reduced alpha diversity compared to AS patients, as well a significantly different microbiome composition (beta diversity) compared to both AS patients and IBD patients. A list of the specific bacterial genera associated with AS, AS-IBD and IBD in the most relevant sample types (terminal ileum and stool) is available in Supplementary Table 1 (*p* < 0.05). The detection of IBD using the findings from the stool microbiome of AS patients was achievable with moderate accuracy (AUC=0.754, 95% CI = 0.588–0.920), similar to the performance of faecal calprotectin (FCP) (AUC=0.752) and better than the DISQ (AUC=0.716). The stool microbiome also accurately distinguished IBD patients from healthy controls (AUC=0.757, 95% CI = 0.643–0.871) (Fig. 1D).

**Fig. 2:**
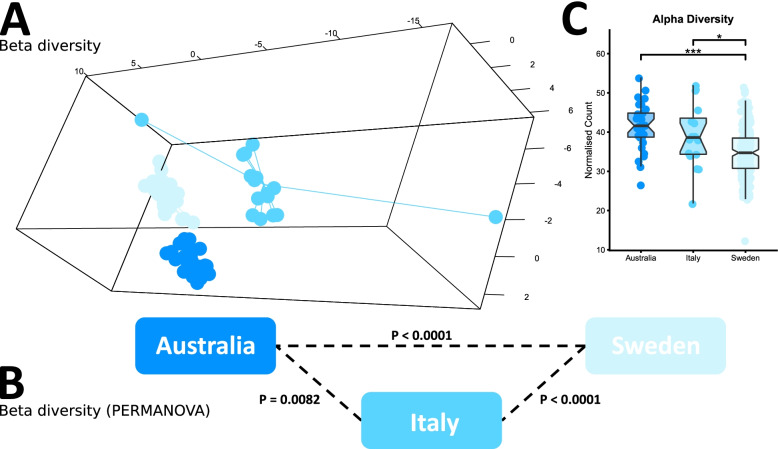
Comparison of stool samples of AS patients from three distinct geographic regions: Australia, Italy and Sweden. **A** sPLSDA visualisation of microbiome composition (beta diversity). **B** PERMANOVA significance testing of beta diversity. **C** Comparison of species richness (alpha diversity)

The composition of the AS microbiome detectable in stool was also compared across three geographic regions, Australia, Italy and Sweden (Fig. 2). As expected, significant differences in both alpha and beta diversity were observed, with AS patients from each region demonstrating unique microbiome composition. Furthermore, Swedish AS patients exhibited a less diverse microbiome overall (alpha diversity). A list of the bacterial taxa enriched or depleted in Italian and Swedish samples, relative to Australian samples is available in Supplementary Table 2. Whilst the sample collection, handling and sequencing, and bioinformatic and statistical analysis was consistent for these samples, a vast array of technical (e.g. duration of storage) and environmental variability (e.g. diet) exists between the cohorts, highlighting the challenges of comparing and interpreting AS microbiomes across different studies.

**Fig. 3:**
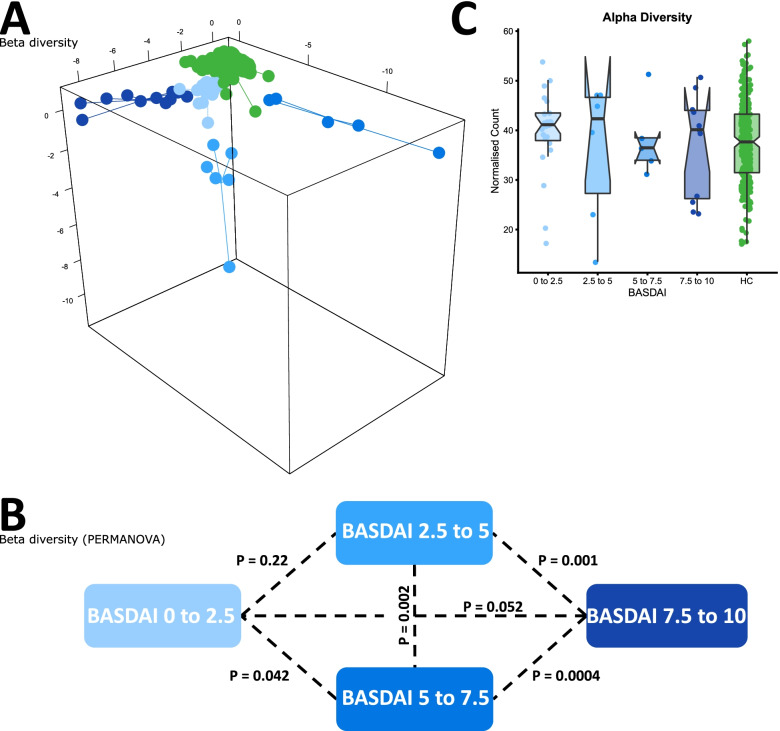
Comparison of microbiome composition in the Australian cohort, sampled from terminal ileum, rectum, right colon and stool, whilst accounting for repeated sampling. Composition was measured according to BASDAI. **A** sPLSDA visualisation of microbiome composition (beta diversity) according to BASDAI. **B** PERMANOVA significance testing of beta diversity according to BASDAI. **C** Comparison of species richness (alpha diversity) according to BASDAI

**Fig. 4:**
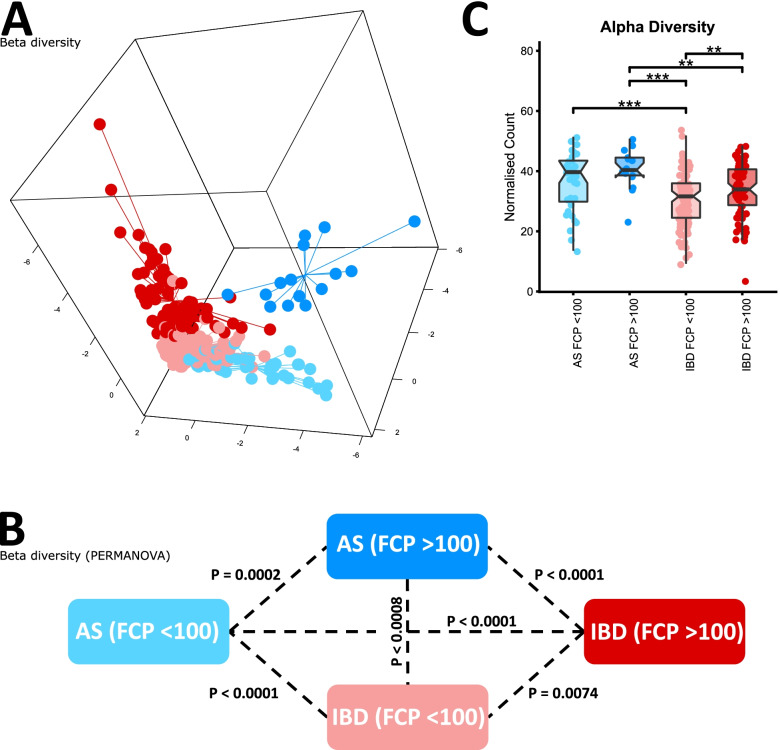
Comparison of microbiome composition in the Australian cohort, sampled from terminal ileum, rectum, right colon and stool, whilst accounting for repeated sampling. Composition was measured according to FCP levels. **A** sPLSDA visualisation of microbiome composition (beta diversity) according to FCP level. **B** PERMANOVA significance testing of beta diversity according to FCP level. **C** Comparison of species richness (alpha diversity) according to FCP level

To investigate potential associations between the gut microbiome and disease characteristics of AS, we studied Australian intestinal biopsies from several sites as well as stool samples and excluding patients not undergoing TNFi treatment. Significant differences in beta diversity according to BASDAI (Fig. 3) were observed, however no significant correlation with *Dialister* was observed, contrary to previous findings [22]. Consistent with previous results, we also noted a significant difference in beta diversity for AS patients with elevated FCP (>100 μg/mg) (Fig. 4). The same effect was also noted for IBD patients in terms of beta diversity, as well as a notable increase in alpha diversity for patients with FCP greater than 100 μg/mg (Fig. 4C), potentially reflecting an increase in the number of inflammatory bacterial taxa.

Previous study of the AS stool microbiome using a GA-map™ Dysbiosis Test in the Swedish cohort revealed an association between dysbiosis and elevated faecal calprotectin levels, but no such association was observed for disease activity assessed by BASDAI [25]. In the current study, when considering changes in the overall microbiome composition, as opposed to association with individual species as conducted in the Swedish study, we note no significant association with FCP (Supplementary Fig. 1). We also noted a significant difference in microbiome composition between individuals with BASDAI 0 to 2.5 versus 5 to 7.5, yet no other BASDAI categories (Supplementary Fig. 1). Consistent with previous reports [25] we note a significant depletion of *Faecalibacterium* in patients with elevated FCP, but we also note an enrichment of several potentially inflammatory genera. A full list of differentially abundant taxa according to BASDAI and FCP are available in Supplementary Tables 3 and 4, respectively.

**Fig. 5:**
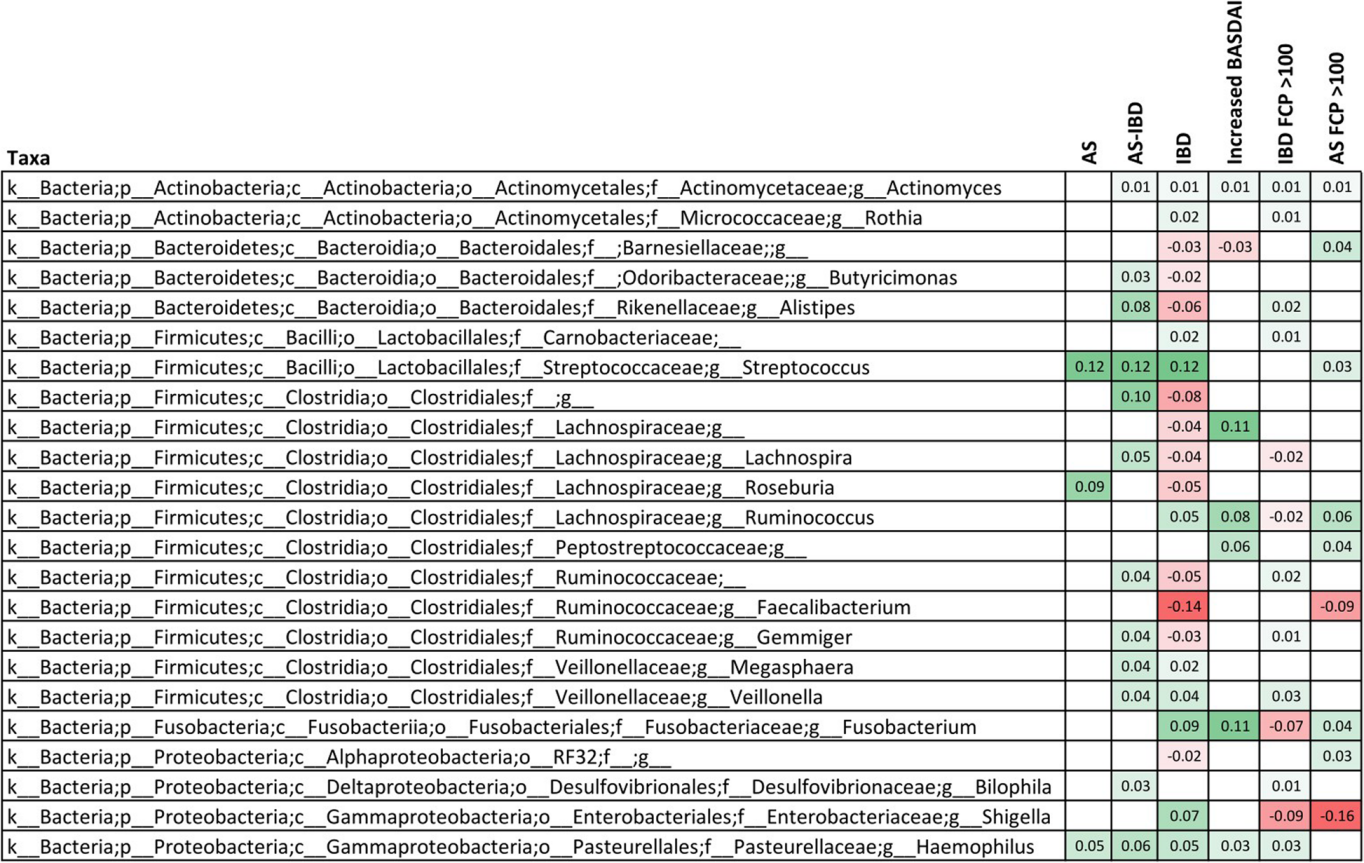
Bacterial genera significantly associated (*p* < 0.05) with multiple clinical attributes. The numbered boxes correspond to the abundance coefficient, with enrichment displayed in green and depletion displayed in red

Bacterial genera which were significantly enriched or depleted in at least two of the following six categories are summarised in Fig. 5: AS patients, AS-IBD patients, IBD patients, patients with increased BASDAI, and AS or IBD patients with FCP >100 μg/mg. The potentially pathogenic genera *Streptococcus* and *Haeomophilus* were noted to be enriched in AS patients, IBD patients, and AS patients with concomitant IBD (AS-IBD). Also of note, *Haeomophilus* was also found to be enriched in patients with increased BASDAI and IBD patients with elevated FCP. Other than these two genera, no other similarities were noted between diseases. Four genera were noted to be enriched in IBD patients and IBD patients with elevated FCP, and *Lachnospira* was noted to be depleted in both IBD and IBD patients with elevated FCP.

## Discussion

Using a standardised analysis, this study reconfirmed the occurrence of gut dysbiosis in AS and IBD patients relative to healthy controls, as well as demonstrating a significant difference between the AS and IBD microbiomes. Differences were not only limited to the overall microbial composition (beta diversity), but also the species richness (alpha diversity), with IBD patients exhibiting a significantly less diverse microbiome compared to AS patients (Fig. 1). Clinically evident IBD has been observed in 6–14% [36] of AS patients. In the current study, AS patients with concomitant IBD (AS-IBD) exhibited a further differentiation of the microbiome compared to AS patients, both in terms of the overall composition and by reduced species richness, acknowledging that the sample size for this analysis was small and that these findings need replication. Furthermore, the presence of IBD in AS patients was also able to be identified with moderate diagnostic accuracy (AUC 0.754) in the stools of AS patients. It follows that the differing pathogenicity of AS compared to IBD corresponds with unique microbiome composition, highlighting the potential multifarious function of the gut microbiome.

The AS microbiome has been studied across a variety of geographically and ethnically diverse populations. In the current study, Australian, Swedish and Italian AS patients all exhibited unique microbiomes from each other (Fig. 2). Whilst the array of environmental and technical factors which differentially influence microbiome composition in each region can be numerous and varied, these results highlight the degree of inter-cohort variability occurring in AS studies internationally. Therefore, careful consideration is required when interpreting AS microbiome studies in isolation, and future meta-analysis of multiple cohorts is likely to require standardised recruitment and sampling protocols.

Disease activity, measured by BASDAI and FCP, was found to correlate with microbiome composition for both Australian AS and IBD patients (Figs. 3 and 4), indicating that therapies which serve to normalise the microbiome may be useful in strategy for patient treatment. Association between disease activity assessed by BASDAI and the stool microbiome has also been reported in a study using 16S ribosomal RNA amplicon sequencing of colonic mucosal samples from patients with spondyloarthritis [22], and normalisation of the gut microbiome in patients with AS on TNF-inhibitors and suppressed disease activity was detected in stool samples via shotgun sequencing [24]. IBD patients with elevated FCP exhibited an increase in alpha diversity, potentially highlighting an increase in inflammatory genera*.* Consistent with previous results, elevated FCP was also correlated with a depletion of *Faecalibacterium,* a bacterial genus with known anti-inflammatory properties. Interestingly, these genera were not correlated with elevated FCP in AS patients highlighting differences between the microbiomes associated with these diseases (Fig. 5). The correlation between FCP and microbiome composition was not reflected in Swedish AS patients; similarly, the correlation between BASDAI and microbiome composition was notably weaker. The stronger results for the Australian cohort may be due to the use of intestinal biopsies which captured the mucosal microbiome interfacing with the immune system, as opposed to stool samples which retain a relatively low level of mucosal microbiome amongst a background of transient luminal microbiome.

Comparison of the bacterial genera significantly associated with at least two of the following disease categories was performed: AS patients, AS-IBD patients, IBD patients, patients with increased BASDAI, and AS or IBD patients with elevated FCP (>100 μg/mg) (Fig. 5). Consistent with the observed microbiome differences between AS and IBD patients, many of the differentially abundant genera were not consistent between AS and IBD, except for enrichment of two potentially pathogenic genera, *Haeomophilus* and *Streptococcus*. Interestingly, enrichment of *Haeomophilus* was also associated with increased BASDAI and IBD patients with elevated FCP. Case reports have suggested that *Haemophilus* sp. are associated with reactive arthritis [37–39]. Similarly, it is well established that *Streptococcus* genus infection is associated with reactive arthritis [40], although it is not HLA-B27-associated, and it appears not to be a form of spondyloarthritis. It follows that despite the divergence of the AS and IBD microbiomes, a small number of pathogenic genera may be the shared/common factors initially triggering disease. However, further species- and strain-level characterisation, enabled by whole genome sequencing, is required to properly determine whether the enriched taxa are in fact pathogenic. Other than these, several genera were also depleted in IBD patients and in IBD patients with elevated FCP. These genera included *Actinomyces*, *Rothia*, and *Lachnospira*, a genus within each of the *Carnobacteriaceae* and *Ruminococcaceae* families.

The use of 16S sequencing in the current study not only limited the taxonomic depth from which conclusions were drawn, but it also prevented a suitable measurement of the functional/metabolic capabilities of the microbiome. Multi-omic, systems biology, approaches (incorporating shotgun metagenomics, transcriptomics, metabolomics and proteomics) are increasingly being used to comprehensively interrogate the microbial features associated with disease. Adopting these approaches in future is essential to move beyond the broad correlations noted in 16S sequencing studies and towards identification of precise microbial features and their causative impacts.

In conclusion, consistent with immunological and genetic evidence, the distinct microbiomes of AS and IBD patients indicate that the gut plays a different role in driving disease. However, enrichment of specific pathogenic genera indicates that the presence of shared/common microbial trigger of disease cannot be discounted. Further research utilising contemporary approaches is required to expand upon these initial observations and to identify precisely the microbial features driving different types of disease.

## Supplementary Information

**Additional file 1: Supplementary Figure 1**. Comparison of microbiome composition in the Swedish cohort, consisting of stool samples which were sampled from some patients twice at a five-year interval. Composition was measured according to BASDAI and FCP levels. A. sPLSDA visualisation of microbiome composition (beta diversity) according to BASDAI. B. PERMANOVA significance testing of beta diversity according to BASDAI. C. Comparison of species richness (alpha diversity) according to BASDAI. D. sPLSDA visualisation of microbiome composition (beta diversity) according to FCP level. E. PERMANOVA significance testing of beta diversity according to FCP level. F. Comparison of species richness (alpha diversity) according to FCP level.

**Additional file 2: Supplementary Table 1**. Indicator species for AS, IBS and AS-IBD patients within the Australia cohort. Indicator species for the most relevant sampling sites (stool and terminal ileum). **Supplementary Table 2**. Indicator species in the stools of AS patients across different geographic regions (Australia, Sweden and Italy). **Supplementary Table 3**. Indicator species associated with BASDAI in Australia AS patients. Samples were collected from multiple sites (stool, terminal ileum, right colon and rectum) and repeated sampling was controlled for. **Supplementary Table 4**. Indicator species associated with FCP in Australia AS and IBD patients. Samples were collected from multiple sites (stool, terminal ileum, right colon and rectum) and repeated sampling was controlled for.

## Data Availability

The datasets used and/or analysed during the current study are available from the corresponding author on reasonable request.

## Abbreviations

AS: Ankylosing spondylitis
AUC: Area under the curve
BASDAI: Bath Ankylosing Spondylitis Disease Activity Index
CI: Confidence interval
FCP: Faecal calprotectin
IBD: Inflammatory bowel disease
ROC: Receiver operator characteristic
sPLSDA: Sparse Partial Least Squares Discriminant Analysis
TNFi: Tumour necrosis alpha inhibitor
