# Supplementary material for: Distinctive gut microbiomes of ankylosing spondylitis and inflammatory bowel disease patients suggest differing roles in pathogenesis and correlate with disease activity
Source: Arthritis Res Ther. 2022 Jul 7;24:163. doi: 10.1186/s13075-022-02853-3 (PMC9261041; doi:10.1186/s13075-022-02853-3)
Supplement: Supplementary file 1 — Additional file 1: Supplementary Figure 1. Comparison of microbiome composition in the Swedish cohort, consisting of stool samples which were sampled from some patients twice at a five-year interval. Composition was measured according to BASDAI and FCP levels. A. sPLSDA visualisation of microbiome composition (beta diversity) according to BASDAI. B. PERMANOVA significance testing of beta diversity according to BASDAI. C. Comparison of species richness (alpha diversity) according to BASDAI. D. sPLSDA visualisation of microbiome composition (beta diversity) according to FCP level. E. PERMANOVA significance testing of beta diversity according to FCP level. F. Comparison of species richness (alpha diversity) according to FCP level. [file 13075_2022_2853_MOESM1_ESM.pdf]

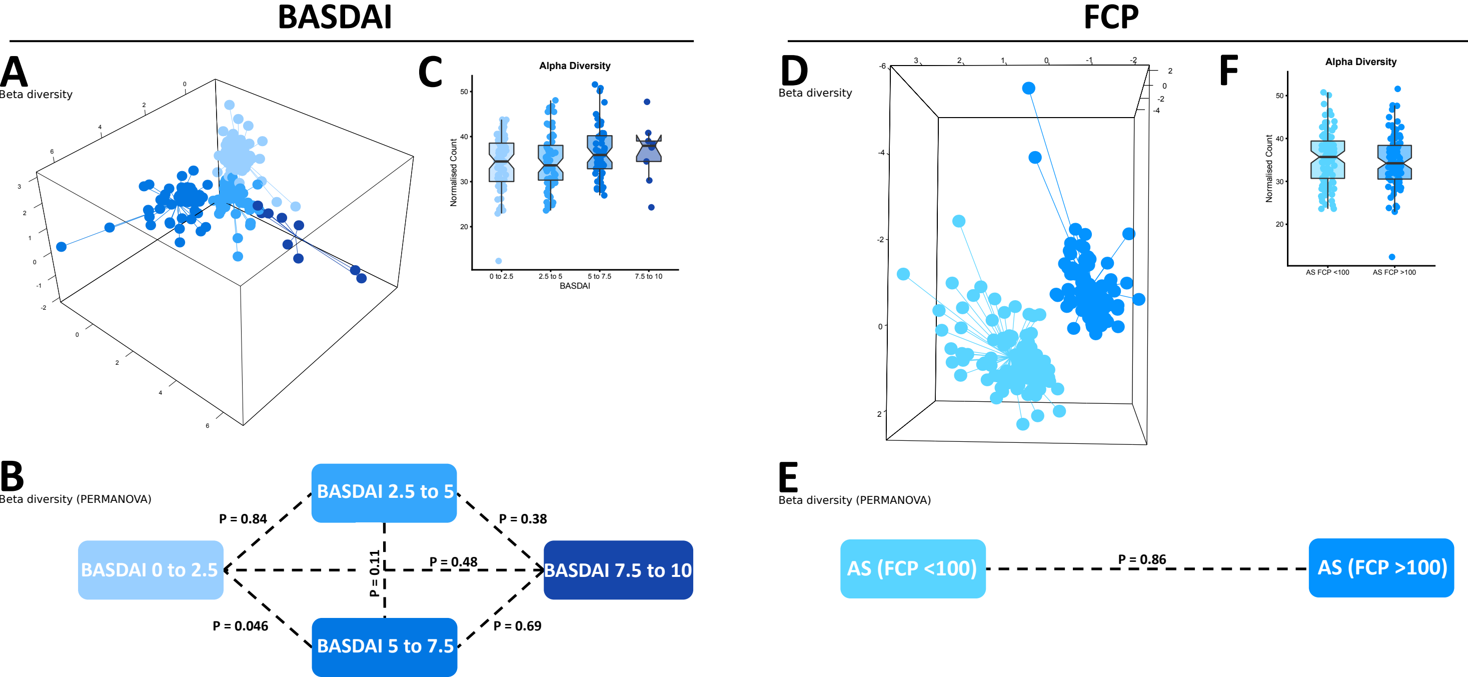
BASDAI
FCP
A
Beta diversity
C
Alpha Diversity
Normalised Count
0 to 2.5
2.5 to 5
5 to 7.5
7.5 to 10
BASDAI
D
Beta diversity
F
Alpha Diversity
Normalised Count
AS FCP <100
AS FCP >100
B
Beta diversity (PERMANOVA)
BASDAI 2.5 to 5
P = 0.84
P = 0.38
BASDAI 0 to 2.5
P = 0.11
P = 0.48
BASDAI 7.5 to 10
P = 0.046
P = 0.69
BASDAI 5 to 7.5
E
Beta diversity (PERMANOVA)
AS (FCP <100)
P = 0.86
AS (FCP >100)
